# Damage to the structural connectome reflected in resting-state fMRI functional connectivity

**DOI:** 10.1162/netn_a_00160

**Published:** 2020-12-01

**Authors:** Anirudh Wodeyar, Jessica M. Cassidy, Steven C. Cramer, Ramesh Srinivasan

**Affiliations:** Department of Cognitive Sciences, University of California, Irvine, CA, USA; Department of Statistics, University of California, Irvine, CA, USA; Department of Allied Health Sciences, University of North Carolina, Chapel Hill, NC, USA; Department of Neurology, University of California, Los Angeles, CA, USA; Department of Cognitive Sciences, University of California, Irvine, CA, USA; Department of Biomedical Engineering, University of California, Irvine, CA, USA

**Keywords:** fMRI, Functional connectivity, Structural connectivity, Stroke, Partial correlation

## Abstract

The relationship between structural and functional connectivity has been mostly examined in intact brains. Fewer studies have examined how differences in structure as a result of injury alters function. In this study we analyzed the relationship of structure to function across patients with stroke among whom infarcts caused heterogenous structural damage. We estimated relationships between distinct brain regions of interest (ROIs) from functional MRI in two pipelines. In one analysis pipeline, we measured functional connectivity by using correlation and partial correlation between 114 cortical ROIs. We found fMRI-BOLD partial correlation was altered at more edges as a function of the structural connectome (SC) damage, relative to the correlation. In a second analysis pipeline, we limited our analysis to fMRI correlations between pairs of voxels for which we possess SC information. We found that voxel-level functional connectivity showed the effect of structural damage that we could not see when examining correlations between ROIs. Further, the effects of structural damage on functional connectivity are consistent with a model of functional connectivity, diffusion, which expects functional connectivity to result from activity spreading over multiple edge anatomical paths.

## Author Summary

Examining the structure to function relationship at damaged edges in the structural connectome due to lesions from stroke we find the following:

•  Partial correlation reflects structural connectome damage in 24% of edges tested.
•  Voxel-to-voxel correlation guided by structural connectivity shows the presence of SC damage in 39% of edges tested.
•  Edges that show modulation when using voxel-to-voxel correlation are consistent with diffusion as a model of functional connectivity.

Our work provides evidence that for pairs of regions that are not directly anatomically connected due to lesions from stroke, signals must be communicated through intermediate region-to-region steps along an anatomical path.

## INTRODUCTION

A large body of literature has demonstrated significant temporal correlations, labeled functional connectivity, in the blood oxygen level-dependent (BOLD) signal between spatially distinct brain regions of interest during rest (e.g., see Greicius, Krasnow, Reiss, & Menon, 2003; Xiong, Parsons, Gao, & Fox, 1999). Resting-state fMRI-BOLD functional connectivity appears to have broad use as a potential biomarker (Drysdale et al., 2017; Liem et al., 2017; Rane et al., 2015) and has been shown to predict task-based BOLD activation (Cole, Ito, Bassett, & Schultz, 2016; Tavor et al., 2016). One critical question that arises is, what mechanism gives rise to resting-state functional connectivity? A potential answer comes from the anatomy-axonal fiber tracts must be the backbone that supports functional connectivity. While the origin of our hypothesis is at the scale of neurons, the fMRI-BOLD and diffusion tensor imaging (DTI) are macroscale (neural mass scale) brain imaging techniques. However, we can expect a persistent statistical relationship across scales that should allow us to relate fMRI-BOLD and DTI. Recently, there have been tools developed (Johansen-Berg & Rushworth, 2009) to map the entire set of fiber tracts and this has been termed as the structural connectome (Sporns, Tononi, & Kötter, 2005). However, conclusively establishing an exact relationship between structural and functional connectivity has proved difficult (Straathof, Sinke, Dijkhuizen, & Otte, 2018).

Identifying the relationship between structural and functional connectivity requires specifying a model of how neural dynamics spread over the axonal fibers. Studies have examined different models of how simple dynamics spread over the structural connectome and their ability to capture the functional connectivity. The simplest model one can assume is that activity spreading over single-edge (defined as a direct structural connectome connection) connections between two distinct regions leads to functional connectivity. Initial examinations of the structural to functional connectivity relationship assuming a single-edge model with simple Gaussian noise dynamics found that structural connectivity was indeed correlated with functional connectivity (see Straathof et al., 2018 for a thorough review, Honey, Kötter, Breakspear, & Sporns, 2007; Koch, Norris, & Hund-Georgiadis, 2002; O’Reilly et al., 2013; Van Den Heuvel, Mandl, Kahn, & Hulshoff Pol, 2009) but at its best, shows shared variance in the range of 60% with the functional connectivity. However, another line of work has examined models that incorporate multi-edge connectivity (Abdelnour, Voss, & Raj, 2014; Avena-Koenigsberger, Misic, & Sporns, 2018; Goñi et al., 2014; Messé, Rudrauf, Giron, & Marrelec, 2015; Mišić et al., 2015), where multiedge connectivity between regions is defined as a connected path over multiple direct structural connections. Incorporating multiedge connectivity improves the variance of functional connectivity (FC) explained by the structural connectivity. By extension, we might expect that we are better able to identify changes in the FC as a function of the absence of single-edge SC connectivity when we reduce the influence of multiedge SC connectivity on the FC.

Patients who have had a stroke nearly always sustain damage to white-matter tracts, thus altering the single-edge SC connectivity. We expect that there is considerable variability in structural connectome damage across patients that arises from the uniqueness of injury (as a function of location and volume) from stroke in each patient. We leverage the resulting heterogeneity in the structural connections of stroke patients to investigate the relationship between structural connections and fMRI functional connectivity. We examined the relationship between structural connectivity and fMRI functional connectivity by using data from patients who have suffered a stroke within a month prior to when data was collected. An estimate of the structural connectome for each patient was obtained using virtual tractography (Kuceyeski, Maruta, Relkin, & Raj, 2013; Pustina et al., 2017) across the pairs of 114 cortical regions of interest (ROIs) from the Lausanne parcellation (Cammoun et al., 2012). Virtual tractography uses a template set of streamlines estimated from healthy subjects intersected with lesion masks for each patient to identify the subset of undamaged streamlines for each patient. This set of undamaged streamlines is used to determine the patient-specific structural connectome. We examine the edge-by-edge relationships of the structural connectivity to fMRI functional connectivity across patients, to identify how differences in structural connectivity across individuals modulates functional connectivity, providing a strong test of the influence of communication over multiedge pathways of the structural connectivity and fMRI functional connectivity.

In this study, we had the unique opportunity to examine which linear model of communication dynamics across the structural connectome best models the impact of damage to axonal fibers from stroke. In a model that expects neural activity to spread only along direct structural connections, functional connectivity would be expected to simply reflect the weights of the structural connectome, that is, damage to the strongest structural connections would have the strongest effects of functional connectivity. In a multiedge model, there is gradient of possible relationships between structural and functional connectivity, depending on extent of information available about the structure. The models relating structural to functional connectivity may be diffusion (Goni et al., 2013; Noh & Rieger, 2004), where communication happens through random walks over the structural connectome. Under diffusion, we expect functional connectivity between two regions to reflect the average number of steps taken by random walkers passing over the structural connectome. Diffusion assumes no global information about the structural connectome, only local information about connection strengths. Another possibility is that functional connectivity navigates the shortest path (Goñi et al., 2014), the strongest weighted path between two regions through the structural connectome. Shortest path routing requires global information about the connectome. We expect that, given the structure to function relationship mediated by multiedge pathways remains consistent across different strengths of an SC connection, then we are able to assess the validity of the hypothesis that multedge pathways are relevant to functional connectivity within a particularly powerful context—that of previously existing edges.

We tested the relationship between structure and function under two analysis pipelines. In the first approach, we estimated correlation and partial correlation by using ROI-averaged signals. Partial correlation is a measure of conditional dependency, that is, it measures, between any two areas, the linear relationship between them when accounting for their relationship to all other areas. We expected partial correlation to better measure information shared over any single-edge path over the SC that is not passing over other edges. We hypothesized that partial correlation would optimally reflect modulation from differences in the structural connectivity across subjects. In our second analysis pipeline, we avoided averaging signals over an ROI by using a voxel-level measure of correlation. Averaging signals over an ROI removes the potential variations that exist at voxel level (Korhonen, Saarimäki, Glerean, Sams, & Saramäki, 2017) and includes voxel-level variations for which we do not possess structural connectivity information. For each pair of ROIs, we used correlations between pairs of voxels connected by a streamline and tested whether damage was linked to altered functional connectivity. By identifying and using only the voxels representing the streamline endpoints of structural connections, we expected that we would be able to better resolve pairs of locations in the fMRI data where we might expect altered functional connectivity. We examined whether we could predict the edges where damage modulated correlations by using models that incorporated only local SC information (diffusion) or a model that incorporated global SC information (shortest path routing). We expected diffusion to be a better model as it is unlikely that neurons store and utilize information about the SC.

## METHODS

### Subjects

Twenty subjects with stroke (with onset 3–26 days prior) admitted to the inpatient rehabilitation facility at the University of California, Irvine, Medical Center participated. All subjects signed informed consent as approved by the Institutional Review Board. Subjects were included if they were older than 18 years with a radiologically confirmed stroke. None of the subjects had other active major neurological or psychiatric conditions or active substance abuse likely to interfere with study procedures along with no prior medical history of cranial surgery. Age varied, with mean ± 1 SD between 57.5 ± 12 years (range 27–79), as did sex (4 female, 16 male) and their extent of arm motor deficits as per their Fugl-Meyer score, which ranged from 10 to 65 (normal score is 66, and higher scores reflect better motor status). Four patients had a hemorrhagic stroke while the other 16 had suffered an ischemic stroke. Further, across all patients, average lesion volume was 23.01 *cm*^3^ (range .8 to 78.1 *cm*^3^). Table 1 shows demographic details about all the subjects involved in our analysis.

**Table 1.** Participant Demographics

Subject ID	Handedness Prior to Stroke	Lesion Site	Lesion Side	Stroke lesion volume (cm^3^)	Stoke Type (I/H)	Sex (M/F)	Time poststroke days	Age (years)	FM Total
S001	L	L pons	L	.79	I	M	14	58	63
S002	R	R basal ganglia	R	36.47	H	F	12	57	57
S003	R	R parietal, R occipital	R	19.87	I	F	11	49	24
S004	R	R basal ganglia	R	2.54	I	F	9	65	14
S005	R	R basal ganglia	R	10.08	H	M	11	40	45
S006	A	L temporal	L	36.97	I	M	5	69	65
S007	R	R basal ganglia, R posterior corona radiata, R temporal lobe	R	15.2	I	M	16	48	13
S008	R	R temporal	R	78.1	H	M	9	50	57
S009	R	R Middle Cerebral Artery, R posterior insula, R parietal operculum	R	69.49	I	M	25	67	26
S010	R	R pons	R	2.18	I	M	18	77	15
S011	R	R periventricular DWM, R PLIC	R	4.77	I	M	9	58	41
S012	L	R basal ganglia	R	68.76	H	M	6	48	17
S013	L	L PLIC	L	3.55	I	M	71	59	8
S014	R	R pons	R	1.36	I	M	17	79	35
S015	R	L pons, L caudal midbrain	L	5.43	H	M	21	51	53
S016	R	L corona radiata, L cerebellar hemisphere, L occipital lobe	L	2.44	I	M	5	67	62
S017	R	R basal ganglia	R	60.38	H	M	26	51	13
S018	R	L corona radiata, R pons, L PLEC	L	1.44	I	M	12	66	53
S019	L	R medulla, cerebellum	R	39.26	I	F	8	27	61
S020	R	L pons	L	1.01	I	M	3	53	52

*Note*. (Legend: L-Left, R-Right, A-Ambidextrous; FM-Fugl-Meyer; I-Ischemic, H-Hemorrhagic; PLIC-posterior limb of internal capsule). FM Total scores range from 0–66, with higher values indicating better arm motor status.

### MRI Processing

Neuroimaging was acquired on a 3.0 T Philips Achieva (Best, the Netherlands) scanner. Anatomical imaging included a high-resolution T1-weighted scan that utilized a three-dimensional magnetization-prepared rapid gradient echo sequence (repetition time (TR) = 8.1 ms, echo time (TE) = 3.7 ms, 150 slices) and a T2-weighted FLAIR scan (TR = 9,000 ms, TE = 120 ms, 33 slices).

We drew lesion masks on T1-weighted MRIs, informed by the corresponding T2 FLAIR images. Masks were generated using techniques (Cassidy, Tran, Quinlan, & Cramer, 2018; Riley et al., 2011) for which reliability has been described previously (Burke et al., 2014). For one subject who was unable to complete an MRI scan, we drew the lesion mask on their corresponding CT scan. Infarct masks were binarized and spatially transformed to MNI space. Lesions on the right were flipped about the midline so that all lesions were in the left side of the brain for analysis purposes. Since motor deficits from stroke in the right hemisphere generally are not different from motor deficits with a left hemisphere stroke, we did not make any adjustments for behavior when flipping lesions.

Subjects completed a resting-state fMRI scan: TR = 2,000 ms, TE = 30 ms, 180 volumes, voxel size = 3.38 × 3.38 × 3 mm^3^. Each patient was instructed to remain still and keep eyes open to avoid falling asleep. Structural and fMRI scans were preprocessed using the CONN toolbox (Whitfield-Gabrieli & Nieto-Castanon, 2012). The initial eight fMRI volumes were discarded. Preprocessing steps included functional slice-time correction, functional realignment (six rigid-body motion parameters), segmentation and spatial normalization to MNI space, functional volume coregistration with structural volumes, functional outlier detection using the artifact removal toolbox (ART), and functional smoothing using a 6-mm^3^ Gaussian kernel. We visually analyzed all coregistrations to ensure quality and accuracy. Functional MRI data underwent denoising procedures to remove unwanted motion, physiological effects, and artifacts. For denoising CONN uses an anatomical component-based noise correction procedure, which includes noise components from cerebral white matter and cerebrospinal areas (Behzadi, Restom, Liau, & Liu, 2007), estimated subject-motion parameters (Friston et al., 1995), identified outlier scans or scrubbing (Power, Schlaggar, & Petersen, 2014), and constant and first-order linear session effects. Finally, global signal regression (Li et al., 2019; K. Murphy & Fox, 2017) was applied prior to bandpass filtering (0.008–0.09 Hz).

### Virtual Tractography

Virtual tractography is a new approach (Kuceyeski et al., 2013; Pustina et al., 2017) to estimating structural connectomes in a stroke population where we can expect structural disconnection from the lesions suffered by patients. This method uses a template of structural connectivity generated from healthy subjects and lesion masks for patients to generate patient-specific structural connectomes. In this way we are able to make maximal use of lesion mask information when we lack diffusion imaging from patients.

For our healthy subject structural connectome template, we used streamlines (representation of the axonal fibers of the brain) generated with deterministic tractography by Yeh et al. (2018) using diffusion imaging from the HCP842 dataset (Van Essen et al., 2013). In this dataset experts vet the streamlines to remove potentially noisy estimates of axonal fibers. We transformed the streamlines generated by Yeh et al. (2018) to the MNI152 template brain accessed from FSL (FMRIB Software Library, https://fsl.fmrib.ox.ac.uk/fsl/fslwiki). Each streamline was approximated by a single 100-point cubic spline using code adapted from the *along-tract-stats* toolbox (Colby et al., 2012). For more details on application of tractography to the HCP diffusion imaging data please refer to Yeh et al. (2018).

We transformed the Lausanne parcellation (Cammoun et al., 2012) of 129 ROIs to the MNI152 template brain and generated a volumetric representation for each ROI using the *easy_lausanne* toolbox (Cieslak, 2015). Estimating virtual tractography for individual patients for subcortical regions was expected to be more difficult than for cortical ROIs as the effects of coregistration errors may be magnified in small volumes. We removed subcortical regions to reduce the parcellation to 114 ROIs. Using the 114 ROIs and the template streamlines we built the adjacency matrix of 114 × 114 ROI pairs. By identifying the streamlines that terminated in each pair of ROIs we were able to create the structural connectome for the Lausanne parcellation. We limited interhemispheric connections to white-matter tracts connecting homologous regions to minimize the contributions of potentially noisy estimates of axonal fibers. Any edge *W*_*ij*_ was defined as the sum over the streamlines biased by the length of the streamline. For ROIs *i* and *j* connected by streamlines *s* of length *l*:

$$W_{ij}=\underset{s_{ij}}{\sum }\frac{1}{l_{ij}}$$ (1)

**Figure 1.**
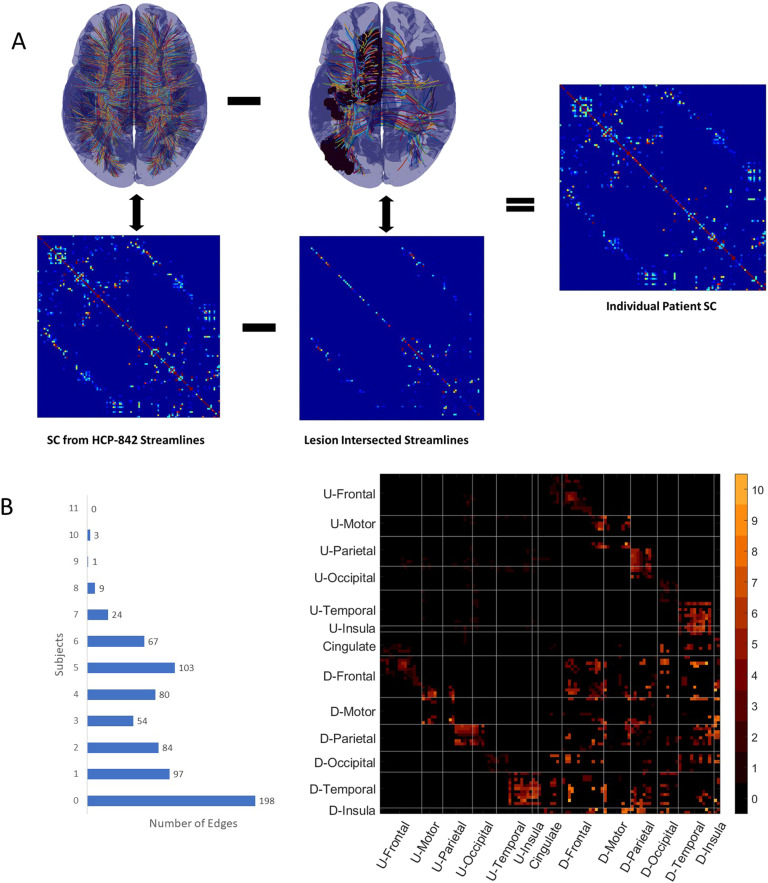
Virtual tractography. (A) we show the pipeline for virtual tractography. We removed from the set of streamlines that represent the undamaged structural connectome (top left) the streamlines intersecting a given patient’s lesion(s), as shown in the top row, middle column. Using the reduced set of streamlines we applied the Lausanne parcellation to get the final structural connectome for that patient (shown on the top row, rightmost column). (B) Edges showing high damage across patients. On the left column we show the number of edges where the 20 patients show high damage (see Virtual Tractography section for definition of high damage). We found that for over 50% of edges there are few (1 or 2) to no patients who sustained high damage to that edge. On the right column we show the structural connectome with edge weight representing number of subjects showing high damage for that edge. In the current analysis we studied all edges (341) where three or more patients had high damage.

Streamlines were weighted by inverse of length to reduce the bias of tractography algorithms toward longer streamlines (Hagmann et al., 2008). We examined the impact of different SC preprocessing strategies—streamline count normalization by ROI sizes, no length bias applied—in our analysis when relevant. The resulting model of structural connectivity shown in Figure 1 is referred to as the structural connectome (SC). We use the abbreviation *SC* to exclusively refer to our model of the structural connectome. All code to perform virtual tractography can be found at https://github.com/wodeyara/Virtual-Tractography.

We intersected the lesion masks (whose creation is described in MRI Processing section) with the template streamlines of the SC. For each patient we are able to identify the template streamlines that are disconnected by the lesion and those that are not based on which streamlines intersect the lesion mask. We remove the streamlines that intersect the lesion mask and use the reduced set of streamlines to define individual structural connectomes. This process is summarized in Figure 1. Each edge for each patient was designated as either high or low damage. High damage indicated that, for that edge, connection strength was less than the median of connection strengths seen for that edge. We show how many edges have high damage across patients in Figure 1. We found that over half of the connections showed high damage in few to no patients. When categorizing the damage in this way, we found that 341 edges (out of 720 total edges) showed high damage in at least three patients. The majority of the edges showing high damage connected frontal (111)—especially motor (48)—and parietal (72) areas in the damaged hemisphere to the rest of the brain. We show these edges by area in Figure 1.

### Estimating FC at the ROI Level

In the first analysis pipeline we estimated fMRI-correlation (fMRI-C) by using conventional linear correlation between ROI-averaged signals. By default, this measure of marginal dependence between areas includes activity shared over multiedge paths over the SC and through common inputs. We can also measure functional connectivity by using partial correlations to define the connection strengths, a measure of the conditional dependency between areas. By estimating the conditional dependency we expect to prioritize activity only shared over single-edge paths and remove the influence of common inputs. We used the adaptive graphical lasso to develop an SC-based model of fMRI-partial correlation or fMRI-PC (Friston, 2011; Marrelec et al., 2006; Reid et al., 2019; Smith et al., 2011). In the process of estimating the model based fMRI-PC, we tested and confirmed (using cross-validation) that the SC is useful in predicting the nonzero elements of the fMRI-PC. We compared at every edge, across patients, whether fMRI-C or fMRI-PC better reflected the impact of damage from stroke.

#### Adaptive Graphical Lasso to Estimate fMRI–Partial Correlation

Partial correlation is estimated using normalized inverse (precision) of the covariance of the fMRI time series. The estimate of the precision from direct inversion tends to be noisy due to an ill-conditioned covariance matrix. To improve the robustness of the precision, it is estimated using the graphical model estimated from the graphical lasso. The graphical lasso (Friedman, Hastie, & Tibshirani, 2008) is a method that has been applied in multiple fields in the past decade, from genomics (Menéndez, Kourmpetis, ter Braak, & van Eeuwijk, 2010)Menéndez, to fMRI functional connectivity (Ryali, Chen, Supekar, & Menon, 2012; Varoquaux, Gramfort, Poline, & Thirion, 2010) and climate models (Zerenner, Friederichs, Lehnertz, & Hense, 2014). In order to apply the lasso, the penalized likelihood function to estimate the precision is defined as follows (Friedman et al., 2008) (where Θ is the covariance):

$$\hat{\Phi }=argmin_{\Phi ≻0}\left(log(det\Phi )+tr(\Theta \Phi )+\lambda \underset{j<k}{\sum }|\Phi_{jk}|\right)$$ (2)

Our purpose was to make use of the lasso while taking advantage of our prior knowledge of the undamaged structural connectome, which we expect are the likely locations of nonzero precision values. We made use of the lasso optimization from QUIC (Hsieh, Dhillon, Ravikumar, & Sustik, 2011) using a matrix penalty term (this process is also called the adaptive lasso (Zou, 2006)) determined by the SC with edges *W* (and *λ*_1_ = *λ*_2_):

$$\hat{\Phi }=argmin_{\Phi ≻0}$$ (3)

$$\left(log(det\Phi )+tr(\Theta \Phi )+\lambda_{1}*\underset{j<k;W_{jk}\in SC}{\sum }|\Phi_{jk}|+\lambda_{2}*\underset{j<k;W_{jk}\notin SC}{\sum }|\Phi_{jk}|\right)$$ (4)

Pineda-Pardo et al. (2014) used the SC weights directly to determine the penalization weights (*λ*), which imposes more structure on the penalization than we assume in our algorithm. We expect that the SC weights will constrain connectivity strengths. However, we do not expect the SC strengths to map directly onto the strength of the precision due to individual differences, as well as variations within individuals across functional brain states. By using an SC template, we are assuming a common SC across participants, and so the expected weight may differ. For this reason we use the binarized SC (with 1s where there exist edges and 0 otherwise) to determine the penalization structure. We estimated the penalization values *λ*_1_ and *λ*_2_ by using cross-validation as described in the next section.

By using the adaptive graphical lasso, we leveraged the information in the SC as a prior for our estimate of the precision. The penalization parameters *λ*_1_ and *λ*_2_ in the graphical lasso determine the set of precision values retained. The output of the lasso when optimizing Equation 2 or Equation 3 is the Gaussian graphical model (GGM). We derive our graph *G* with vertices *V* = 1, 2, … *C* and edges *W*_*est*_ = *G*_*ij*_ = 1, *i*, *j* ∈ *V* from the GGM based on the nonzero values in the precision. The final precision matrix ${\hat{\Theta }}^{-1}$ is estimated under the Gaussian likelihood for the set of edges *W*_*est*_ defined by the graphical model ($\hat{\Phi }$) using the function *ggmFitHtf* (PMTK3 toolbox; (K. Murphy & Dunham, 2008)); which optimizes (unpenalized Gaussian log-likelihood):

$$\tilde{\Phi }=argmin_{\Phi ≻0;|\Phi |>0=G}-\left(log(det\Phi )+tr((\Theta +\delta *I)\Phi )\right)$$ (5)

Since **Θ** (covariance) is frequently rank deficient, we add a small value (*δ*) along the diagonal to make it full rank. We define *δ* as 0.001 times the maximum value along the upper triangle of the covariance.

All of the code used to perform the analysis described above are available at https://github.com/wodeyara/fMRI-PC.

#### Cross-validation to Test SC Model of Functional Connectivity

We tested whether the adaptive graphical lasso produced estimates of the precision that show reduced error relative to applying the graphical lasso, *thereby directly demonstrating the link between structural and functional connectivity*. We split the fMRI data from patients into two halves. Using graphical lasso and adaptive graphical lasso we estimated the precision $\tilde{\Phi }$_1_ on one half of samples of fMRI-BOLD data and estimated the deviance when using this precision as the estimate of the inverse for the covariance of the other half Θ_2_ of the data (and vice versa). Deviance was estimated as:

$$Dev=.5(-log(det{\tilde{\Phi }}_{1})+tr(\Theta_{2}{\tilde{\Phi }}_{1})-log(det{\tilde{\Phi }}_{2})+tr(\Theta_{1}{\tilde{\Phi }}_{2}))$$ (6)

We applied the process of cross-validation over the same range of penalization for *λ*_1_ in the adaptive graphical lasso (3) and for *λ* in the graphical lasso (Equation 2). For the adaptive graphical lasso (Equation 3) we maintained *λ*_2_ (the penalization for non-SC edges) constant at 0.2.

**Figure 2.**
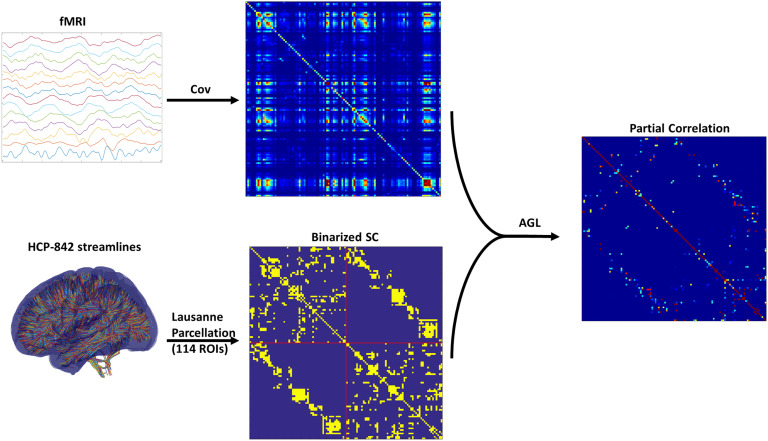
Using the adaptive graphical lasso (AGL) for fMRI-PC. This figure shows the general processing pipeline for applying the AGL to estimated patient fMRI covariance. The binarized undamaged SC is used to bias the penalization used when optimizing under the AGL to estimate the precision. We used the normalized precision to generate the partial correlation (fMRI-EC).

**Figure 3.**
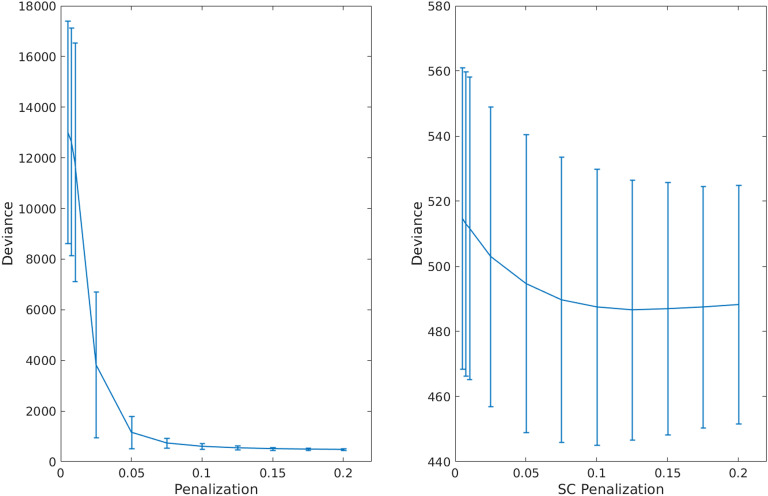
Cross-validation of graphical lasso (GL) and adaptive graphical lasso (AGL). We show performance (cross-validated deviance) across penalization for the GL (on the left) and AGL (right figure). The error bars represent the standard deviation of the deviance across patients. We found that for all but very high penalization the AGL outperformed the GL. Further, the AGL shows minimal cross-validated deviance for an optimal set of SC edges (at an SC penalization of 0.125) while the graphical lasso shows minimum deviance only for very sparse models.

We found that with the graphical lasso a sparser model (fewer connections) is always better (indicated by the decreasing cross-validated deviance). However, when using our adaptive graphical lasso, we find there is an optimal penalty which minimizes the cross-validated deviance and consistently outperforms the graphical lasso see (Figure 3). *This clearly demonstrates the structure-function relationship as the prior of using the SC provides useful information in predicting the fMRI–partial correlation (evidenced by the reduced cross-validated deviance) over and above using a uniform prior.* The results from this analysis are shown in Figure 2. Based on the minimum deviance from cross-validation, we estimated fMRI-PC using the adaptive graphical lasso with the penalization value of 0.125 on the SC edges (i.e., *λ*_1_ from Equation 3) and 0.2 (*λ*_2_ from Equation 3) on the edges not in the SC.

#### Partial Correlation

We estimate partial correlation as the summary statistic for the Gaussian graphical model. We estimate the partial correlation (for any two areas *x* and *y* and estimated precision $\tilde{\Phi }$):

$$PC_{xy}=\left|\frac{\Phi_{xy}}{\sqrt{\Phi_{xx}*\Phi_{yy}}}\right|$$ (7)

We use *PC* as our estimate of the single-edge functional connectivity and refer to it as the fMRI-PC. We use this partial correlation similarly to the correlation matrix when estimating the impact of SC damage. This is discussed in section Estimating Functional Connectivity at the ROI Level.

#### Modeling Impact of SC Damage

We transformed the correlation and partial correlation across subjects by taking their absolute value and applying the Fisher r-to-z transform. For the partial correlation we added a small value (10^−4^) to make all values nonzero since the gamma distribution is undefined for 0. Let *C*_*ijk*_ represent the transformed correlation between ROIs *i* and *j* for subject *k* and *P*_*ijk*_ the transformed partial correlation. We treat the *C*_*ijk*_ and *P*_*ijk*_ as gamma-distributed variables (see Supporting Information Figure S1 for gamma distribution fit) because the values are positively valued and tend to be skewed toward the positive axis of the real line. Using this notation, *C*_*ij*:_ and *P*_*ik*:_ represent the response vectors for gamma-(GLSM) generalized linear models by using a log link function. We used the stratified SC damage as a predictor for the GLM (represented as *SC*_*ij*:_). Thus the models built for each structural connection *W*_*ij*_ can be represented as (*E* is the expectation):

$$ln(E(C_{ij:}))=\beta_{0}+\beta_{1}*SC_{ij:}$$ (8)

$$ln(E(P_{ij:}))=\beta_{0}+\beta_{1}*SC_{ij:}$$ (9)

The fMRI-C and partial correlation edge by edge models were multiple comparisons corrected using false detection rate (FDR) correction with *α* set to 0.05.

### Estimating Functional Connectivity at the Voxel Level

To calculate the functional connectivity estimates (correlation and partial correlation), we initially averaged over all voxels within each ROI, which averaged the fMRI data over both voxels that contributed to the structural connectivity count and voxels that did not. We identified the voxel-based analysis as a means of reducing this shortcoming of averaging the fMRI signal over an ROI and instead hoped to identify the fMRI-C over single-edge paths (as a function of the streamlines), which we expected would be a better correlate of structural connectivity from the neural signals recorded by fMRI within each ROI.

The Lausanne estimate of any structural connectome edge aggregates structural connections by counting the number of streamlines that connect a pair of ROIs. The termination points of streamlines can be approximated as a voxel within each ROI, and only a subset of voxels serve as termination points. Other voxels within the ROI have different streamline patterns, that is, they either connect to different ROIs or we do not have connectivity information for them entirely.

**Figure 4.**
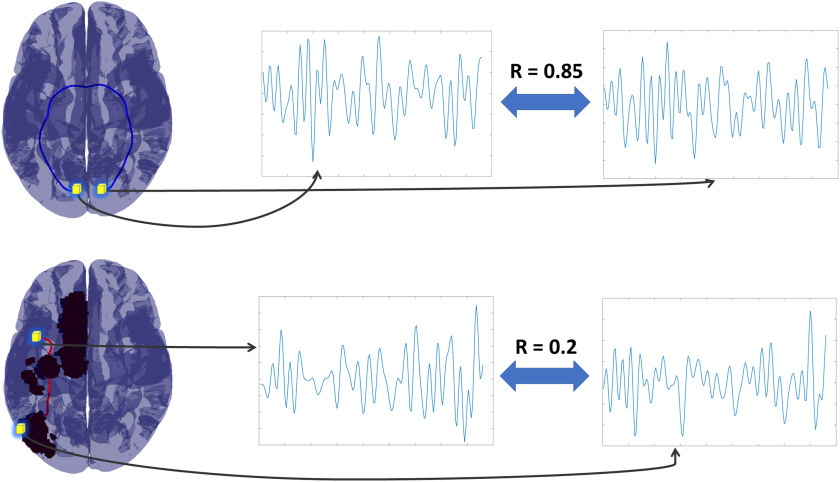
Streamline functional connectivity (sFC). We show how sFC was calculated for two streamlines in two different patients. For each streamline the termination points were identified as fMRI voxels (shown as the yellow shaded cubes in the brain in the leftmost column above), and we estimated the correlation between these voxels. The same was done for both connected (top row where the color blue indicates the streamline remained connected) and disconnected streamlines (bottom row, where the color red indicates a disconnected streamline). Arrows in the figure identify the fMRI time series for the voxel termination points with the correlation between each pair of voxels reported between the pair of time series. We repeated this process for all streamlines for each subject.

We ran a fine-grained streamline-by-streamline analysis as we predicted we would be better placed to then identify changes in the concomitant functional connectivity. Using the unique pairs of voxel termination-points for every streamline, we estimated the fMRI streamline functional connectivity (sFC) using the correlation of that pair of voxels (see Figure 4). This resulted in, for every edge of the SC matrix for 114 ROIs, a set of fMRI correlations derived for streamlines across subjects that were intact, and a set of fMRI correlations derived for streamlines across subjects that were disconnected.

Finally, we tested different models of communication to identify which model better predicted which edges were likely to be modulated. We expected that modulated edges were more likely to be part of multiedge pathways. So for this delimited set of edges we compared models of functional connectivity generation (diffusion and shortest path routing) over the structural connectome that use multiedge pathways but require different extents of information about the structure.

#### Modeling Impact of SC Damage

We took the absolute value of the streamline correlations and applied the Fisher r-to-z transform. We again assumed that the correlation *sC*_*ijkl*_ for ROIs *i* and *j* and streamline *k* for each subject *l* is gamma distributed (see Supporting Information Figure S2 for gamma distribution fit) and used this as the response variable. We used an indicator variable *sSC*_*ijkl*_ that defined for ROIs *i* and *j* whether streamline *k* for a specific subject *l* was disconnected (1) or connected (0) as the dependent variable. We built gamma GLMs across subjects for each edge:

$$ln(E(sC_{ij::}))=\beta_{0}+\beta_{1}*sSC_{ij::}$$ (10)

We corrected for multiple comparisons using FDR with *α* set to 0.05.

#### Communication Models of Functional Connectivity

We tested using models of functional connectivity whether we could estimate which edges showed the impact of damage when using fMRI-sFC. We simulated functional connectivity weights (FC weights) given models of diffusion (Goni et al., 2013) and shortest path (Goñi et al., 2014). Diffusion assumes that neural activity randomly propagates across the connectome (with the probability of an edge being traversed being proportional to its ROI relative weight) when passing between any two ROIs. In contrast, given a shortest path communication process, it’s assumed that neural activity takes the shortest weighted path between any two ROIs. Note that a shortest path process depends on global information of the structural connectome, while diffusion needs only local information of the structural connectome. As the baseline case we also examine single-edge routing, the process of activity spreading across direct SC edges.

We estimate functional connectivity under a diffusion process using the *mean_first_passage_ time.m* function from the Brain Connectivity Toolbox (Rubinov & Sporns, 2010). This function estimates the “mean first passage time” (MFPT) between any two ROIs *a* and *b*—the average number of weighted steps for a random walker going from *a* to *b*. We standardize (subtract the mean and divide by the standard deviation) the MFPT across nodes and make it symmetric for analyses. A lower value of MFPT for an edge indicates it is more rapidly reached by a random walker, indicating a higher expected functional connectivity. We estimate functional connectivity under a shortest path routing process by using the betweenness centrality (Rubinov & Sporns, 2010) for every edge. The edge betweenness centrality (BC) calculates how many shortest paths use any edge in the structural connectome. We standardized the BC for our analysis. A higher value of the BC indicates greater use by a shortest path routing process and thus a higher expected functional connectivity. Finally, we directly used the standardized SC weights as a third possible predictor of functional connectivity—increased structural connectivity weights directly correspond to increased functional connectivity. All simulated functional connectivity strengths were estimated using an undamaged weighted structural connectome.

The set of edges (represented by *W*_*dmg*_) where SC damage influenced our connectivity measure (fMRI-sFC) was assumed to be a binomially distributed random variable (with 1s when damage significantly influenced connection strength and zero otherwise). Using Wilcoxon rank-sum tests, we first examined whether there was a significant difference in diffusion (using MFPT), shortest path (using BC), and *SC*_*weight*_ between edges where stroke-related damage altered connection strength versus edges where it did not. When there was a significant difference between the two groups for multiple predictors, we built a logistic regression model as follows (assuming for this example case that all three—MFPT/BC/*SC*_*weight*_—showed significant differences):

$$logit(E(W_{dmg}))=\beta_{0}+\beta_{1}*MFPT+\beta_{2}*SC_{weight}+\beta_{3}*BC$$ (11)

## RESULTS

### ROI Level Analysis Shows that SC Damage Modulates fMRI-PC But Not fMRI-C

**Figure 5.**
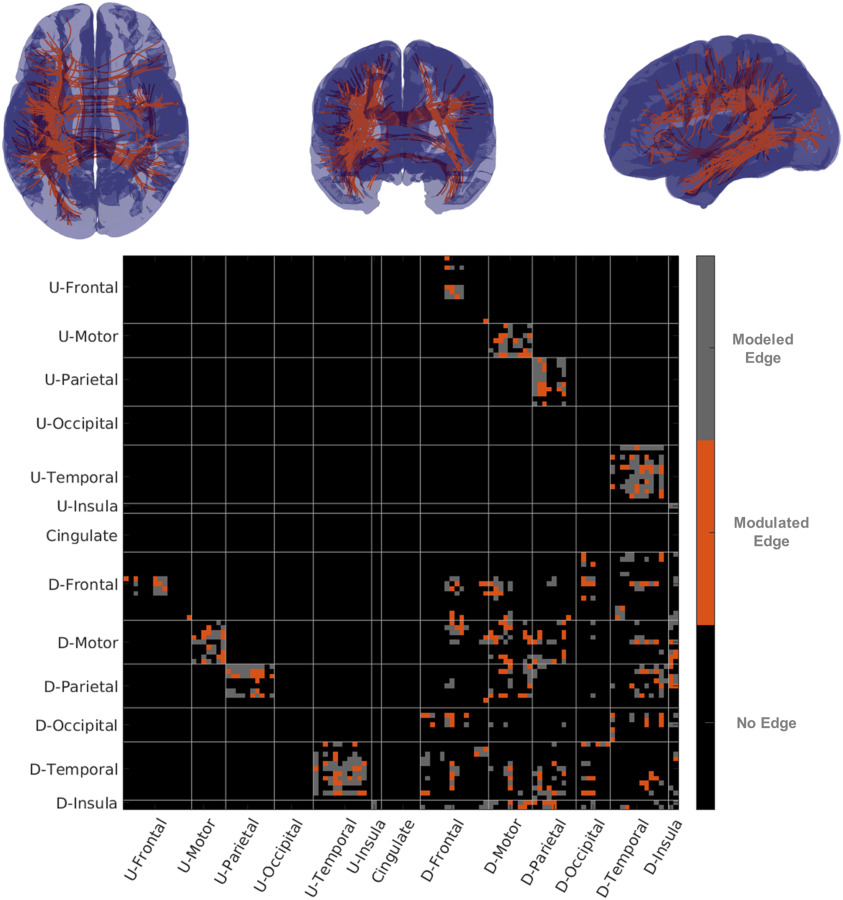
fMRI–partial correlation and structural connectivity. The top row of figures show representative streamline connectivity (every edge where damage influenced fMRI-PC has one streamline shown) for which damage influenced partial correlation. In the figure on the lower row we show a matrix with edges modeled in gray and edges where fMRI-PC was modulated in orange. U-Areas represent sets of areas in the undamaged (contralesional) hemisphere, and D-Areas represent groups of areas in the damaged (ipsilesional) hemisphere.

We averaged the fMRI data over each ROI of the Lausanne parcellation. Using this data we computed the fMRI-C and fMRI-PC between the 114 cortical ROIs. We estimated the fMRI-PC using the adaptive graphical lasso. We examined the models built between all pairs of appropriate SC edges with the response being the partial correlation/correlation, and the predictor is the presence of white-matter damage. We initially only examined the set of edges where at least three patients showed the presence of high damage. When examining fMRI-C we found only 1 edge out of 341 was significantly modulated by damage. When using the fMRI-PC (Figure 5) on the other hand, 24% (80/341) of edges were significantly modulated by damage (applying an FDR correction for multiple comparisons). We also tested how many edges were modulated by damage when we altered the number of patients who needed to show the presence of high damage before we modeled that edge. When we set our threshold at least five patients showing high damage, we again only found 1 edge showing the presence of damage in fMRI-C and 25% (52/207 edges) using fMRI-PC. Finally, when we set the threshold to at least seven patients, in fMRI-PC 22% (8/37) edges showed the presence of damage and no edges in the fMRI-C showed the presence of damage. For an examination of all possible thresholds and number of edges significant, please see Supporting Information Table S1.

### Voxel-Level Analysis Shows that SC Damage Modulates fMRI-sFC

**Figure 6.**
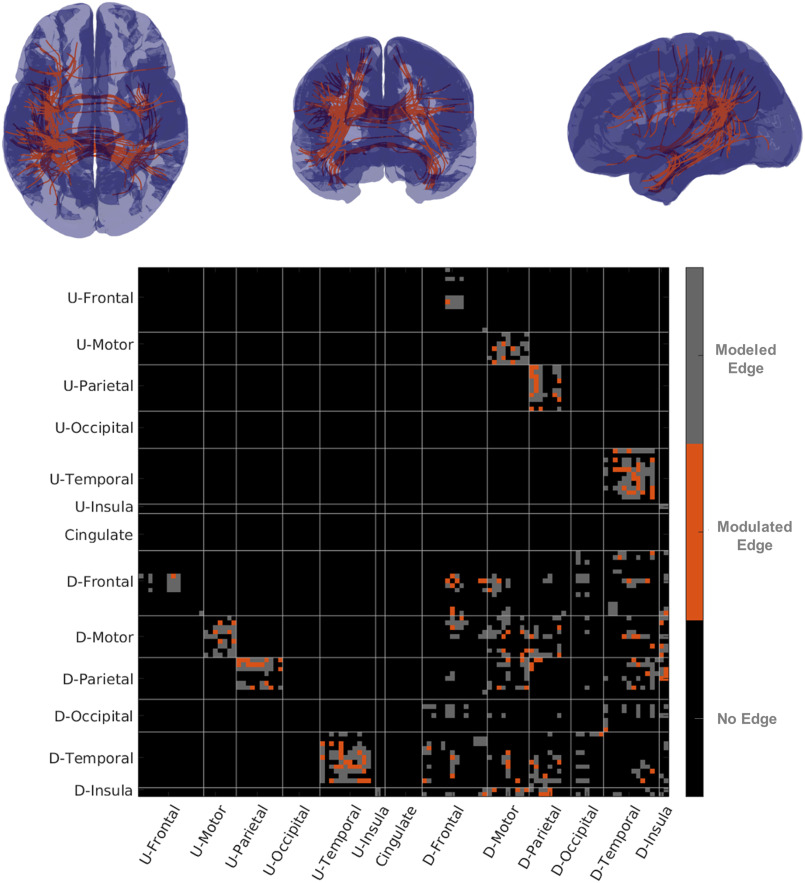
Streamline functional connectivity and structural connectivity. In the top row figures show representative streamline connectivity for edges for which damage influenced fMRI-sFC. On the bottom row is a matrix with edges modeled in gray and edges where sFC was modulated in orange. U-Areas represents sets of areas in the undamaged (contralesional) hemisphere and D-Area represents groups of areas in the damaged (ipsilesional) hemisphere.

Similar to the analysis we used when we averaged fMRI data over ROIs, we examined the streamline-based functional correlation. The fMRI-sFC analysis was also organized using the 114 ROI Lausanne parcellation. We built gamma GLMs between fMRI-sFC and the presence of streamline disconnection for each edge where at least three patients showed high damage. We found that we could identify modulation from disconnection in the fMRI-sFC in 39% (132/341) of the edges we modeled (see Figure 6). When at least five patients showed high damage, 38% (79/207) of edges had sFC that was influenced by streamline disconnection, and when over seven patients showed high damage then 15 out of 37 (41%) edges had sFC modulated by disconnection.

We examined the breakdown of where damage influenced fMRI-PC and fMRI-sFC by looking at groups of ROIs (for a breakdown by pairs of regions, see Supporting Information Tables S2 and S3). For the fMRI-sFC, we found that damage could be detected in 42% (37/89) of damaged motor connections, 52% (16/31) of damaged occipital connections, and 39% (37/94) of parietal connections. On the other hand for the fMRI-PC, we found that temporal (24%, 35/144 edges), parietal (28%, 26/94 edges) and the insula (25%, 8/32) reflected the impact of damage the most often. For the sFC, intrahemispheric connections (42.5%, 77/181 of edges modeled) were more easily identified relative to interhemispheric connections (31%, 50/160 of edges modeled). However, we saw this (weakly) reversed for the fMRI-PC where interhemispheric connections were more easily identified 27% (43/160) relative to 20% (37/181) of intrahemispheric connections modeled.

### Relevance of SC Edges Modulating fMRI-sFC

Using the undamaged structural connectome, we estimated the MFPT and edge betweenness centrality (BC) between all pairs of nodes. We extracted and standardized the SC weights (*SC*_*weight*_), MFPT, and BC for the 341 edges where we modeled the impact of damage. We treated these values as FC weight estimates and examined if they predicted the set of edges where we could estimate the presence of damage in fMRI-sFC. Our analysis approach directly compares these FC estimates against one another, while traditional analyses comparing different communication models examine them across the entire connectome, including in places where there isn’t a direct structural connection.

We found that all three FC weight estimates (MFPT, BC, and *SC*_*weight*_) were significantly different (Wilcoxon rank-sum test, *p* < 0.05) between edges where fMRI-sFC was modulated or unmodulated by structural damage. To see their relative influence, we used them together in a logistic regression model. The response for the model was a vector of length 341×1 with zeroes for nonmodulated edges (fMRI-sFC did not significantly differ between damaged and undamaged streamlines) and ones for modulated edges (fMRI-sFC significantly differed between damaged and undamaged streamlines). We found that MFPT (*β*_*MFPT*_ = −0.63 ± 0.4, 95*%* CI; *p* = 0.003) had a stronger influence on the odds of an edge showing the impact of damage than the betweenness centrality (*β*_*BC*_ = 0.27 ± 0.32, 95% CI, *p* > 0.1) and the raw SC weights (*β*_*SC*_*weight*__ = 1.5 ± 1.75, 95% CI, *p* > 0.1). The negative value indicates that for a unit increase in (the standardized) MFPT, which indicates a reduced FC weight estimate, the odds of damage impacting the fMRI-sFC are multiplicatively reduced by 0.52.

We reestimated the three FC weight estimates (MFPT, BC, and *SC*_*weight*_) when streamline counts for each edge were not biased by length and also when we normalized the streamline count by the product of ROI sizes for each pair of ROIs connected by an edge. When the SC edges were determined by the streamline counts directly (no normalization or biasing by length), we again found the MFPT (*β*_*MFPT*_ = −0.72 ± 0.31; *p* = 7.75 × 10^−6^) to have a stronger influence on the odds relative to betweenness centrality (*β*_*BC*_ = 0.22 ± 0.32, *p* > 0.1) and the SC weights (*β*_*SC*_*weight*__ = 0.24 ± 0.27, *p* = 0.09) directly. Finally, when we normalized streamline counts by the product of ROI sizes, we found that MFPT continues to have a stronger influence on the odds, *β*_*MFPT*_ = −0.77 ± 0.3 (*p* = 1.3 × 10^−6^), while BC (*β*_*BC*_ = 0.05 ± 0.26; *p* > 0.1) and SC weights (*β*_*SC*_*weight*__ = 0.27 ± 0.3; *p* = 0.09) aren’t significant predictors.

## DISCUSSION

We found that a measure of functional connectivity averaged over ROIs that emphasized information shared over single-edge paths (partial correlation) was modulated in 24% of edges with structural connectivity damage. Further, using the SC as a prior when estimating the fMRI–partial correlation showed reduced predictive error to using a uniform prior. In parallel, we found that functional connectivity at the voxel level showed the impact of structural connectivity damage in 39% of edges modeled, with the edges modulated under damage better predicted by a multi-edge model of functional connectivity that uses only local information about the SC (diffusion). Together, when we considered BOLD signal at both the voxel scale and the ROI scale, we saw how differences in structural connectivity were linked to changes in functional connectivity. Thus, we found that, in the brain, communication between neural masses is mediated through anatomical connections. For pairs of regions that are not directly anatomically connected due to lesions from stroke, signals must be communicated through intermediate region-to-region steps along an anatomical path.

The current study found that damage does not effectively modulate fMRI functional connectivity as measured by correlation between ROIs. Partial correlation, as we hypothesized, does show the impact of damage, though in a circumscribed set of edges. Past work employing partial correlations to examine structural connectivity to functional connectivity relationships have been few. In humans, partial correlation was used to study the voxel-level functional connectivity and its relationship to the structural connectome (Horn, Ostwald, Reisert, & Blankenburg, 2014). In this study (Horn et al., 2014), the authors found that while a significant relationship existed between voxel-level functional connectivity and the structural connectome, the correlation between them was comparable to the correlation between voxel-level functional and structural connectivity. Another study (Park, Eo, & Park, 2017) found that structural connectome edge strength was correlated with the variability of partial correlation across multiple sessions. Further, these authors (Park et al., 2017) found that the structural connectome edge strength was less correlated with the fMRI-C, suggesting partial correlation is a more robust link to structure. Finally, in a mouse study of the structure-to-function link (Huebner et al., 2017), demyelination led to reduced partial correlations for long-range connections. Our work is in line with the latter studies, since damage is clearly linked to fMRI-PC rather than to fMRI-C.

Functional MRI data has millimeter-scale spatial resolution of neural activity. However, when estimating the fMRI-C and fMRI-PC, we removed this source of variability by averaging over voxels in an ROI, which served to reduce the influence of noise, but also averaged over heterogeneity in the neural activity present within an ROI. Critically, averaging over voxels in an ROI combines fMRI data from voxels for which we have structural information with those for which we do not have this information. By using fMRI-sFC, we tried to examine fMRI data potentially at the scale at which we have structural connectivity information. One other study followed a similar approach (Skudlarski et al., 2008) and found that when only examining pairs of voxels with a direct structural connection, and estimating a pseudo functional connectivity from the structure by using all polysynaptic paths, they saw a correlation of 0.26 between the pseudo functional connectivity and empirical functional connectivity. When all voxels were used to correlate their estimate of the pseudo functional connectivity with the empirical functional connectivity, they saw a correlation of 0.18 across 5,000 voxels (note that 61% of voxels were disconnected, i.e., pseudo functional connectivity was 0). Horn et al. (2014) looked at the correlation between structural and functional connectivity at the voxel level. They found the correlations were much weaker, 0.046 at 40,000 voxels; however, they examined all-to-all voxel structural connections and voxel correlations. In our work, when examining voxel-level correlations between pairs of voxels connected by a-streamline, we saw that the fMRI-sFC was modulated by streamline disconnection across 39% of the edges modeled. Collectively, these findings suggest that choosing voxels by using streamlines may be a good approach to constrain the data space for fMRI functional connectivity to that accessible to tractography applied to diffusion-weighted imaging when the goal is to link functional connectivity to structural connectivity.

Several studies have examined what communication models most tightly link the functional connectivity to structure. In a macaque study, the amygdala was inhibited using designer receptors exclusively activated by designer drugs (DREADDs), which led to a reduction in functional connectivity between amygdala and the rest of the brain (Grayson et al., 2016). The decrease in functional connectivity could be predicted by a model of neural communication over the structural connectome that incorporated all walks (potentially repeated multiedge pathways) across the network, requiring each node to possess only local information, captured by the *communicability*. Using communication models that incorporate all walks over the network can be reexpressed as a graph eigenmode comparison problem (Tewarie et al., 2020). Comparing the structure and function graph eigenmodes, Atasoy, Donnelly, and Pearson (2016) and Robinson et al. (2016) demonstrated a tight relationship between the structure and function. Goñi et al. (2014) directly tested the relationship between the information about the connectome needed by communication between two ROIs to occur over the shortest path and the functional connectivity, finding that lesser information needed to find the shortest path led to higher functional connectivity. Other works (Abdelnour et al., 2014; Messé et al., 2015; Mišić et al., 2015; Osmanlıoğlu et al., 2019) have used generative models that use only local information about the connectome and have shown that functional connectivity is well explained by these models. In related work to our analysis, Griffis, Metcalf, Corbetta, and Shulman (2020) showed that using an estimate of shortest paths in a damaged structural connectome could predict altered functional connectivity. However, they did not compare different communication models. We examined whether specific communication models incorporating local information (diffusion) or global information (shortest paths) about the SC could be distinguished based on the set of edges where damage was linked to altered fMRI-sFC. We found that edges with rapid diffusion are more likely to show the fMRI-sFC modulation by damage, as compared to edges that were central to the shortest paths between ROIs. By limiting our tests between models to the actual edges of the structural connectome we provide new evidence for diffusion as a relevant model of communication for functional connectivity. Finally, a crucial analysis for future studies is the examination of nonzero functional connectivity at edges of anatomical path lengths greater than 1 and their relationship to behavior and potential clinical relevance.

For a short interval immediately following stroke (on the scale of weeks), past studies have demonstrated an increased capacity for neural plasticity (Cramer et al., 2011; T. H. Murphy & Corbett, 2009). Neural plasticity after a stroke can occur through new structural changes or new functional organization of surviving neural elements, or both. In our work, we have examined a group of patients in the subacute interval (between 3 days to 1 month) when we expected that while there is increased capacity for plasticity, the effects of plasticity are as yet not expressed in all patients. We have treated every patient as having the same structure to function relationship at every edge; however, this expectation may not be true. Our inability to see the influence of damage at every edge modeled likely reflects this additionally heterogeneity in the data. Future work will hopefully examine the relationship between structure and function in even more localized temporal intervals (say within the first week poststroke) or during intervals far beyond initially elevated periods of plasticity to identify its impact (3 or more months poststroke (Cassidy et al., 2020)). These studies will reveal in greater clarity the structure-function relationship immediately poststroke, and the relationship after the effects of neuroplasticity are expressed.

Our work is relevant to understanding the relationship between structure and function in injury, pursuant to the caveats arising from our expectations of the influence of plasticity. The fact that a large percentage of edges showed that the functional connectivity disruption will be distinguishable in the presence of structural damage indicates that there is some constancy in the relationship between structure and function under structural injury. There has been simulation work (Alstott, Breakspear, Hagmann, Cammoun, & Sporns, 2009; Cabral, Hugues, Kringelbach, & Deco, 2012; Irimia & Van Horn, 2014; Váša et al., 2015) that found changes to global network properties, and global functional dynamics (decrease in FC distant from lesion) in the presence of circumscribed lesion damage. These past studies have predominantly assumed nonlinear associations between areas, and further, assumed that the structure-to-function relationship is unaltered under damage, except for a change in connection strengths. Future empirical and theoretical work would be needed to assess whether global network consequences are simply a superposition of local SC-FC edge relationships (a linear relationship) or result from nonlinear relationships. Given our knowledge of neuronal communication, nonlinear relationships are expected at the neuronal scale; however, the SC-FC relationship may be well approximated at the macroscale by a linear relationship. Our work (as well as that of Griffis et al., 2020) suggests weak evidence in favor of a linear relationship between SC and FC, even under damage to the SC.

Major limitations in our work are linked to issues with tractography first, and secondly, with virtual tractography. There are difficulties in tractography linked to overlapping fiber bundles that make it hard to identify correct bundle endpoints, and strict correction of incorrect streamlines can rapidly lead to large numbers of false negatives (Maier-Hein et al., 2017). Our decision to remove nonhomologous interhemispheric connectivity may have introduced a few false negatives. By examining the structural connectome against a template, virtual tractography ignores individual variability in white matter, both healthy and in relation to damage from a lesion. While estimating structural connectivity damage with diffusion imaging for patients of stroke is challenging (Karnath, Sperber, & Rorden, 2018), it remains plausible (Gleichgerrcht, Fridriksson, Rorden, & Bonilha, 2017). Further, there is the potential for additional burden of cerebrovascular risk factors within the set of subjects studied. As we do not know of their actual structure, our virtual tractography only approximates the entire extent of structural damage. The results presented here made the best possible use of the stroke lesion imaging available via structural MRI in conjunction with a pre-existing DTI atlas to obtain an estimate of white-matter damage. It remains up to future work to examine the relationship of structure to function by using individual estimates of structural connectomes drawn directly from diffusion-weighted imaging. Another methodological limitation of this study is the use of a common space (MNI template) to run our analyses. Also, we use streamline end points to estimate fMRI-sFC. However, there will be cases where these end points are distant from gray matter. In our analysis procedure, when this occurs, there is no fMRI signal at the voxels, and the streamline does not contribute to discrimination of when damage influences fMRI-sFC. Finally, we have used a short interval of fMRI data for our analysis (180 samples), and, optimally, future work will examine data collected for longer periods of time.

Despite these caveats, we found that we could see the influence of damage in 24% of SC edges when using partial correlation and in 39% of SC edges when using streamline functional connectivity. Our work provides impetus to more deeply probe the structure-function relationship, particularly the influence of multiedge pathways on functional connectivity both in healthy brains and in injury.

## SUPPORTING INFORMATION

Supporting information for this article is available at https://www.doi.org/10.1162/netn_a_00160.

## AUTHOR CONTRIBUTIONS

Anirudh Wodeyar: Conceptualization; Formal analysis; Methodology; Software; Visualization; Writing - Original Draft; Writing - Review & Editing. Jessica M. Cassidy: Conceptualization; Data curation; Funding acquisition; Investigation; Methodology; Writing - Review & Editing. Steven C. Cramer: Conceptualization; Funding acquisition; Methodology; Supervision; Writing - Review & Editing. Ramesh Srinivasan: Conceptualization; Funding acquisition; Methodology; Resources; Supervision; Writing - Review & Editing.

## TECHNICAL TERMS

Diffusion: Communication between neural masses in distinct areas happens through activity passing by random walks over the structural connectome.
Partial correlation: Summary statistic of conditional dependency, i.e., it measures, between any two areas, the linear relationship between them when accounting for their relationship to all other areas.
Virtual tractography: Using lesions masks and structural connectome template to estimate structural connectomes in a stroke population where we can expect structural disconnection from the lesions suffered by patients.
Graphical lasso: Optimization scheme used to estimate the inverse of covariance while optimizing the L1 norm, thereby pushing small values of the precision to zero.
Gaussian graphical model: A network defining the nonzero conditional dependency relationships between variables, here, brain areas.
Deviance: Cross-validated error in estimating the precision derived from the log-likelihood for Gaussian graphical models.
Generalized linear model: An extension to linear regression that allows us to model response variables that, given the predictors, are not gaussian distributed, here, gamma distributed.
fMRI streamline functional connectivity: Using the termination points of streamlines to determine the pair of voxels for which we use the fMRI data to estimate correlation.
(Edge) Betweenness centrality: A measure of how many shortest paths over a network use an edge.
