# Supplementary material for: Damage to the structural connectome reflected in resting-state fMRI functional connectivity
Source: Netw Neurosci. 2020 Dec 1;4(4):1197–218. doi: 10.1162/netn_a_00160 (PMC7781612; doi:10.1162/netn_a_00160)
Supplement: Supplementary file 1 [file netn-04-1197-s001.pdf]

Wodeyar, A., Cassidy, J. M., Cramer, S. C. & Srinivasan, R. (2020). Supporting information for “Damage to the Structural Connectome reflected in resting state fMRI Functional Connectivity.” *Network Neuroscience*, 4(4), 1197–1218. https://doi.org/10.1162/netn_a_00160

Supplementary Information - Damage to the Structural Connectome reflected in resting state fMRI Functional Connectivity

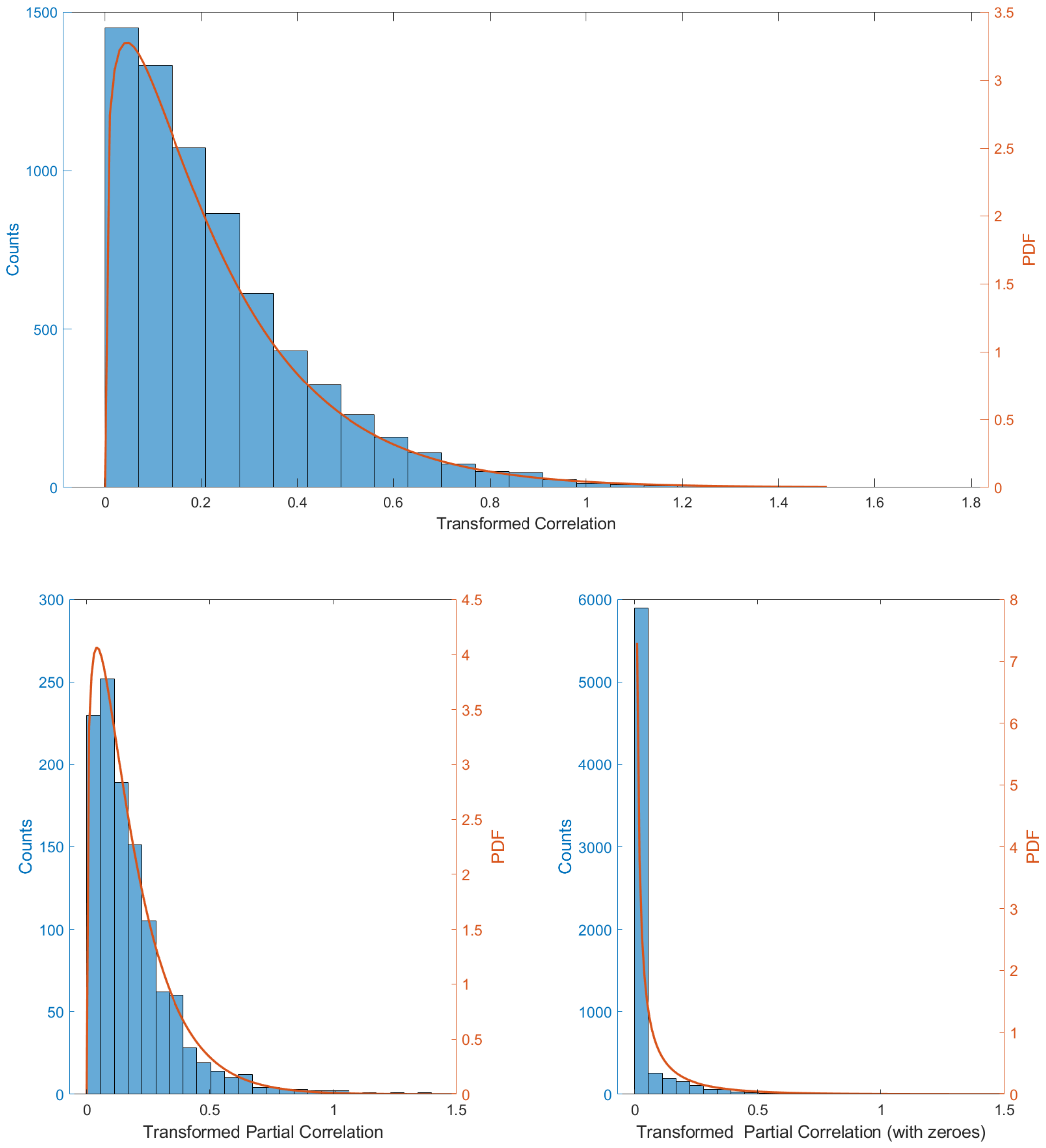

Supp Figure 1 – Gamma Distribution Fits Correlation and Partial Correlation: We show how data are well matched by a gamma distribution fit to the correlation (fMRI-C) and the partial correlation (fMRI-PC) estimated across pairs of ROIs. In the lower row we show the difference between examining the partial correlation without and with zeros. Optimally, given more subjects, we would fit a binomial-gamma hurdle model but we have far too few data points to fit more than one parameter. Thus, with the motivation of maximizing power we fit only a gamma GLM to the partial correlation data at each edge.

Wodeyar, A., Cassidy, J. M., Cramer, S. C. & Srinivasan, R. (2020). Supporting information for "Damage to the Structural Connectome reflected in resting state fMRI Functional Connectivity." *Network Neuroscience*. Advance publication. https://doi.org/10.1162/netn_a_00160

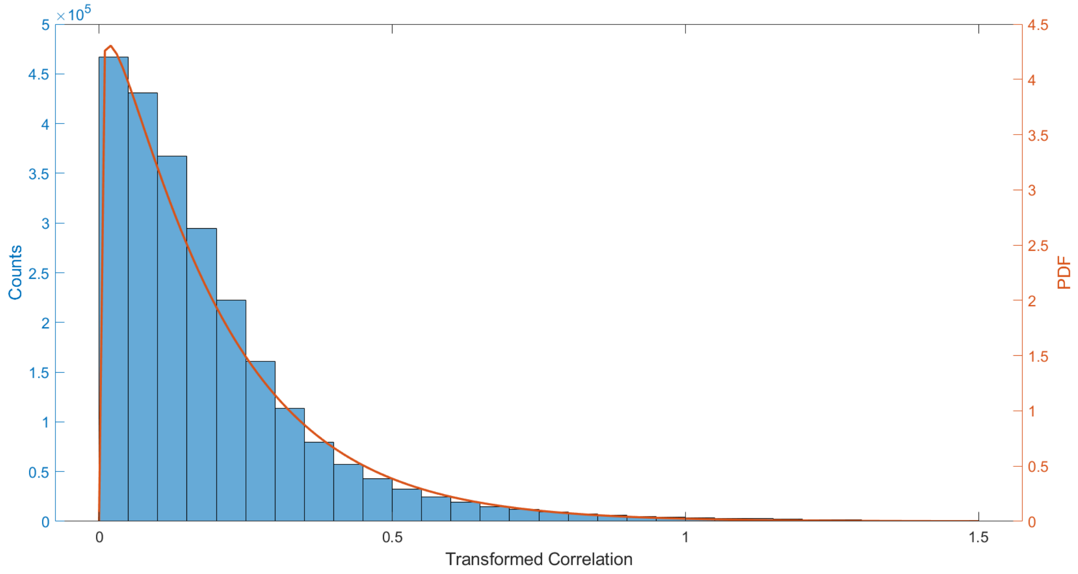

Supp. Figure 2 - Gamma Distribution Fit to Voxel Correlation: We show that the voxel to voxel correlations are well fit by a gamma distribution.

	Correlation		Partial Correlation		Streamline Correlation	
Number of Subjects (threshold)	Edges Significant	Num Edges Tested	Edges Significant	Num Edges Tested	Edges Significant	Num Edges
3	1	341	80	341	132	341
4	1	287	70	287	111	287
5	1	207	52	207	79	207
6	0	104	23	104	38	104
7	0	37	8	37	15	37
8	0	13	3	13	6	13
9	0	4	2	4	2	4
10	0	3	2	3	1	3

Supp. Table 1 - Edges Significant as a Function of Number of Subjects: We show the number of edges identified as significant (i.e. there was a significant difference in the functional connectivity between undamaged and damaged SC edges) at different thresholds on the number of subjects showing high damage. See Methods section for details.

Wodeyar, A., Cassidy, J. M., Cramer, S. C. & Srinivasan, R. (2020). Supporting information for “Damage to the Structural Connectome reflected in resting state fMRI Functional Connectivity.” *Network Neuroscience*. Advance publication. https://doi.org/10.1162/netn_a_00160

Area One	Area Two	Edges Modulated	Edges Modeled	Ratio
'U-Frontal'	'D-Frontal'	1	17	0.06
'D-Frontal'	'D-Frontal'	4	7	0.57
'U-Motor'	'D-Motor'	6	32	0.19
'D-Frontal'	'D-Motor'	2	15	0.13
'D-Motor'	'D-Motor'	3	13	0.23
'U-Parietal'	'D-Parietal'	14	39	0.36
'D-Motor'	'D-Parietal'	4	20	0.20
'D-Parietal'	'D-Parietal'	2	2	1.00
'U-Temporal'	'D-Temporal'	22	70	0.31
'D-Frontal'	'D-Temporal'	4	24	0.17
'D-Motor'	'D-Temporal'	4	10	0.40
'D-Parietal'	'D-Temporal'	4	23	0.17
'D-Occipital'	'D-Temporal'	1	13	0.08
'D-Temporal'	'D-Temporal'	1	4	0.25
'D-Frontal'	'D-Insula'	1	6	0.17
'D-Motor'	'D-Insula'	3	10	0.30
'D-Parietal'	'D-Insula'	4	7	0.57

Supp. Table 2 - Number of Edges Significant For All Pairs of ROIs using fMRI-PC: We show above, for any pair of ROIs that have at least one edge that showed modulation of the fMRI-PC, how many edges were tested and the ratio between edges that show up as significant and total edges tested. (Note that D- stands for Damaged-Side and U- stands for Undamaged-side)

Wodeyar, A., Cassidy, J. M., Cramer, S. C. & Srinivasan, R. (2020). Supporting information for “Damage to the Structural Connectome reflected in resting state fMRI Functional Connectivity.” *Network Neuroscience*. Advance publication. https://doi.org/10.1162/netn_a_00160

Area One	Area Two	Edges Modulated	Edges Modeled	Ratio
'U-Frontal'	'D-Frontal'	7	17	0.41
'D-Frontal'	'D-Frontal'	4	7	0.57
'U-Motor'	'D-Motor'	13	32	0.41
'D-Frontal'	'D-Motor'	6	15	0.40
'D-Motor'	'D-Motor'	8	13	0.62
'U-Parietal'	'D-Parietal'	14	39	0.36
'D-Frontal'	'D-Parietal'	1	4	0.25
'D-Motor'	'D-Parietal'	10	20	0.50
'D-Frontal'	'D-Occipital'	8	12	0.67
'U-Temporal'	'D-Temporal'	21	70	0.30
'D-Frontal'	'D-Temporal'	8	24	0.33
'D-Motor'	'D-Temporal'	3	10	0.30
'D-Parietal'	'D-Temporal'	7	23	0.30
'D-Occipital'	'D-Temporal'	8	13	0.62
'D-Temporal'	'D-Temporal'	3	4	0.75
'D-Motor'	'D-Insula'	5	10	0.50
'D-Parietal'	'D-Insula'	5	7	0.71
'D-Temporal'	'D-Insula'	1	4	0.25

Supp. Table 3 - Number of Edges Significant For All Pairs of ROIs using fMRI-sFC: We show above, for any pair of ROIs that have at least one edge that showed modulation of the fMRI-sFC, how many edges were tested and the ratio between edges that show up as significant and total edges tested. (Note that D- stands for Damaged-Side and U- stands for Undamaged-side)

Wodeyar, A., Cassidy, J. M., Cramer, S. C. & Srinivasan, R. (2020). Supporting information for “Damage to the Structural Connectome reflected in resting state fMRI Functional Connectivity.” *Network Neuroscience*. Advance publication. https://doi.org/10.1162/netn_a_00160
